# Lifestyle behaviors among clients of diet centers in Jeddah, Saudi Arabia: exploring physical activity and sleep quality beyond dietary programs

**DOI:** 10.3389/fpubh.2026.1782131

**Published:** 2026-03-27

**Authors:** Elaf Babtain, Manal Mansoury, Arwa Turkistani

**Affiliations:** Department of Food and Nutrition, King Abdulaziz University, Jeddah, Saudi Arabia

**Keywords:** diet centers, lifestyle behaviors, physical activity, Saudi Arabia, sleep quality

## Abstract

**Background:**

Diet centers that offer packaged, tailor-made dietary programs have emerged in the market in Saudi Arabia. However, it has not been examined to what degree enrolling in such diets is associated with adequate physical activity and sleep quality.

**Objective:**

To explore if clients following diet programs by Diet centers in Jeddah, Saudi Arabia, engage in a good lifestyle by performing physical activity and sleeping well, and follow the diet plan as a routine.

**Methods:**

A cross-sectional study was carried out with 200 adults (19–45 years) enrolled in 18 diet centers. Participants were divided into groups of low-calorie (LC), Mediterranean (MD), high-protein (HP), low-calorie ketogenic (LCK), and control groups. Structured interviews, as well as the Pittsburgh Sleep Quality Index (PSQI), the International Physical Activity Questionnaire (IPAQ), and three-day 24-h dietary recalls, were used to gather data. Statistical analyses were conducted with SPSS.

**Results:**

There were statistical differences among the dietary groups in energy intake, macronutrient composition, sleep quality, and physical activity (*p* < 0.001). The MD group showed the best sleep quality with 95% good sleep and a low median PSQI score of 3, with the LCK group reporting the worst sleep performance and the highest short sleep duration (10% reported a good sleep and a median PSQI of 8). Sleep duration was normal for 92.5% of the MD group *vs.* 27.5% of the LCK group. A significant association was obtained between physical activity and sleep, with 50.7% of moderately active participants reporting good sleep quality vs. 20.4% of inactive participants (*p* = 0.001). Furthermore, 42.5% of the LCK group were inactive, compared to 22.5% of the MD and 5% of the HP group (*p* < 0.001).

**Conclusion:**

In this sample of diet center clients, adherence to dietary plans was high, but engagement in complementary lifestyle behaviors varied significantly by diet type. Incorporation of structured physical activity and sleep health education in diet center programs may be associated with better lifestyle change and positive health outcomes.

## 1 Introduction

Saudi Arabia’s nutrition environment is experiencing a rapid shift in response to increasing urbanization, changing lifestyle habits, and demand for convenience-based food solutions (1). In this evolving marketplace, diet centers have become important sources of structured weight-management programs. These centers provide personalized, ready-to-eat meals designed to meet clients’ caloric, macronutrient, and health needs. This method has gained popularity as the national overweight/obesity rates increase (2). These centers establish themselves as holistic, time-saving options for those in need of weight management or following healthy lifestyle.

The demand for such services is reinforced by the country’s high prevalence of obesity and metabolic disease. Recent national surveys show the obesity rate among the population aged 15 and above is 23.1%, while 45.1% of individuals in this age group are classified as overweight (3). Hence, it is well documented that obesity contributes substantially to the burden of type 2 diabetes, cardiovascular disease, and other non-communicable diseases (4). Thus, structured dietary interventions have become a common approach for people seeking easy and convenient ways to improve their health (5).

However, while diet is essential for weight management and improving metabolism, evidence consistently shows that diet alone is not enough to achieve the best and lasting health benefits. Studies across diverse populations show that combining dietary modification with regular physical activity (PA) yields significantly greater reductions in body weight, visceral fat, glycated hemoglobin (HbA1c), and cardio-metabolic risk markers than diet-only interventions (6–8). Physical activity enhances insulin sensitivity, promotes lean body mass preservation, increases resting metabolic rate, and improves cardiovascular fitness (9). Sleep quality forms the third pillar of a healthy lifestyle, interacting bidirectionally with both diet and physical activity. Poor sleep duration and quality are related to increased appetite, higher intake of energy-dense foods, impaired glucose tolerance, and lower motivation for physical activity (10). This results in hormonal and metabolic imbalances (11, 12). Conversely, diets high in fiber, omega-3 fatty acids, and micronutrients such as magnesium and tryptophan are associated with improved sleep architecture (13). Meta-analyses showed that short sleep (7 h/night) increases the risk of overweight and central obesity (14, 15).

Physical inactivity and poor sleep are major public health concerns in Saudi Arabia. Such deficiencies might limit the effect of diet-only interventions. National data indicated that the percentage of the total Saudis that did not engage in physical activity (i.e., ≥ 150 min per week) among Saudi adults aged ≥15 y was 82.60% (16). Physical inactivity is cited as responsible for 6 per cent of the disease burden associated with coronary heart disease, 7 per cent of type 2 diabetes, 10 per cent of breast cancer, and 10 per cent of colon cancer (17). Sleep issues are common too: the previous studies of Saudi communities found poor sleep problems in 37.5% of adults (18).

These findings indicate that the typical lifestyle context of Saudi adults—including clients of diet centers—may limit the success of dietary interventions when physical activity and sleep behaviors are not concurrently addressed. Despite the strong theory and evidence supporting a complete, multi-part approach to weight management, no published study has investigated whether clients of diet centers in Saudi Arabia follow physical activity recommendations and good sleep habits along with their assigned dietary plans. Existing research on lifestyle behaviors in the Kingdom has focused on the general population, university students (19), clinical samples, or individuals attending fitness facilities (20, 21), but not on those subscribing to structured, ready-made diet programs. Understanding whether diet-center clients engage in adequate physical activity and maintain healthy sleep patterns is essential for informing strategies aimed at optimizing weight outcomes, improving metabolic health, and designing more comprehensive, evidence-based programs tailored to the Saudi context. Addressing this gap will help determine whether diet programs should integrate physical activity and sleep-health support to enhance long-term effectiveness. This study aims to examine whether clients following diet programs from diet centers adopt a healthy lifestyle by engaging in physical activity and maintaining good sleep habits.

## 2 Methods

### 2.1 Study design and participants

A cross-sectional study was conducted among attendees of diet centers from October 15, 2024, to February 21, 2025. A convenience sampling strategy was used to select diet centers. The study included 18 diet centers from different regions of Jeddah (North, South, East, and West) in Saudi Arabia, ensuring regional representation. Invitations to participate in the study were sent online to the selected centers to obtain authorization from center managers. The advertisement described the study objectives and participation criteria. A control group was recruited through advertisements on social media platforms, and eligible respondents were subsequently enrolled. The study included males and females aged 19–45 years who had attended the selected diet centers for at least 3 months. Exclusion criteria included obesity (BMI > 30 kg/m^2^) and the presence of chronic diseases, as obesity and chronic conditions can affect sleep quality by altering metabolism and sleep–wake cycles, and excess body weight may have direct physical effects on sleep (22).

Exclusion criteria included obesity (BMI ≥ 30 kg/m^2^) and the presence of chronic diseases. Although individuals with obesity constitute a substantial proportion of clients attending diet centre’s, obesity was excluded to minimize confounding related to obesity-associated physiological and metabolic alterations known to independently affect sleep quality, physical activity capacity, and circadian regulation. Individuals with obesity are more likely to experience sleep disturbances such as obstructive sleep apnea, altered sleep architecture, and reduced physical activity tolerance, which could obscure associations between dietary patterns, physical activity, and sleep behaviors (23–26). By restricting the sample to underweight, normal-weight, and overweight individuals, this study aimed to examine lifestyle behaviors beyond diet in a relatively metabolically homogeneous group, thereby improving internal validity and allowing clearer interpretation of associations between dietary program type, physical activity level, and sleep quality. Those diagnosed with sleep disorders, as well as pregnant and lactating women, were also excluded. Using G*Power version 3.1, the sample size was calculated as follows: A large effect size (0.5) was assumed, with a significance level (*α*) of 0.05. A minimum sample size of 128 participants was needed to obtain a statistical power of 0.80.

A total of 200 participants were included in the study (*n* = 40 per group). Participants were categorized into diet groups based exclusively on the named dietary program they were enrolled in and receiving prepared meals from at their respective diet centers. The first group followed a low-calorie diet (LC), the second group followed a Mediterranean diet (MD), the third group followed a high-protein diet (HP), the fourth group followed a low-calorie ketogenic diet (LCK), and the fifth group served as a control group with no dietary restrictions. All participants consumed diets based on their estimated daily energy requirements, as determined by a nutritionist according to individual health status, except for the LC group, whose total daily energy intake was reduced by 300–750 kcal. Similarly, participants in the LCK group followed a caloric restriction of 300–750 kcal, with carbohydrate intake limited to approximately 5% of total energy. The HP group was characterized by a higher protein intake, accounting for 30%–35% of total energy intake. The MD group followed a diet rich in fiber, fruits, vegetables, and olive oil. The control group received no specific dietary instructions or restrictions.

Ethical approval was obtained from the Research Ethics Committee, KAU (Reference No 133-24). Authorization was also obtained from the diet centers to recruit participants, and the centers assisted in advertising the study among their clients. Written informed consent was obtained from each participant before enrolment, containing information about the study’s aims, purposes, procedure, and their right to accept, refuse, or withdraw participation. All methods were performed in accordance with the relevant ethical guidelines and regulations.

### 2.2 Data collection tool

Participants were interviewed face-to-face using structured questionnaires. The questionnaire was adapted from previous studies (27, 28). For content validity, the pre-translated questionnaires in Arabic were used and modified to ensure cultural compatibility. A pilot study was conducted to validate the questionnaire, involving 20 randomly selected attendees from diet centers. Then, their feedback was integrated into the questionnaire. The Alpha Cronbach’s coefficient was 0.85 for the IPAQ and 0.86 for the PSQI, which is considered appropriate. The purpose of the pilot study was to identify potential errors or barriers optimize the questionnaire’s clarity and coherence, and verify the data collection time and method.

The first section of the questionnaire collected demographic information, including age, gender, employment status, educational level, and health status. It also recorded the duration of the diet regimen (in months) and anthropometric measurements, including weight (kg) and height (m). Then, the BMI was calculated to classify the participants as underweight (<18.5 kg/m^2^), normal weight (18.5–24.9 kg/m^2^), or overweight (25.0–29.9 kg/m^2^) (29).

The second section included the PSQI (27). Subjective sleep quality, sleep latency, length, habitual sleep efficiency, sleep disruption, use of sleeping medication, and daytime dysfunction are the components of the PSQI, which evaluates sleep quality and disturbances during 1 month. Each component is scored from 0 to 3, yielding a global score ranging from 0 to 21, the poor sleep quality was defined as score of ≥5, while good sleep quality score of <5. Moreover, sleep time was classified as “Long” (≥9 h), “Normal” (7–8 h), “Short” (5–6 h), and “very short” (<5 h) (27). The validated Arabic version of the PSQI was used in this study. The Arabic PSQI has demonstrated good internal consistency and construct validity in Arabic-speaking populations, including Saudi samples, with reported Cronbach’s alpha values ranging from 0.77 to 0.83 (30).

The third section included the IPAQ Arabic version to provide estimates of physical activity level (28). The IPAQ form assesses the frequency, duration (in minutes), and intensity of physical activities lasting at least 10 minutes over the previous 7 days, as measured by the metabolic equivalent of task (MET) value. Each domain within the questionnaire has a specific MET value for each type of physical activity. The MET score, calculated by multiplying the duration and frequency of specific activities, was used to determine overall physical activity level. Scores below 600 METs indicated physical inactivity, scores between 600 and 1,500 METs indicated moderate activity, and scores above 1,500 METs indicated high physical activity (31).

The fourth section collected dietary intake data using three non-consecutive 24-h food recalls, including two weekdays and one weekend day, as recommended in dietary assessment methodology in order to assess participants’ adherence to the prescribed diet plan (32, 33). The recalls were used to estimate total energy intake and macronutrient consumption (protein, carbohydrate, and fat) in grams, using Food Processor software. Because protein, carbohydrate, and fat were reported in grams, energy (in calories) was calculated by multiplying protein and carbohydrate (g) by 4 and fat (g) by 9, respectively. Each of these three categories was divided by total energy to determine the percentage daily intake of each nutrient.

### 2.3 Statistical analysis

The data were statistically analyzed using SPSS version 27 (IBM, 2020). The qualitative data were shown as frequencies and relative percentages. The count data were presented as mean ± SD. The Chi-square test was also used to compare qualitative variables. Quantitative variables were correlated with Pearson’s correlation coefficient. Furthermore, for comparisons of quantitative variables from more than two groups of normally distributed data, the ANOVA F-test combined with the LSD *post hoc* test was used, while the Kruskal-Wallis test combined with Dunn’s post hoc test was used for comparison of non-normally distributed data. The significance level was set at a *p* value of <0.05.

## 3 Results

### 3.1 General characteristics of the studied groups

Table 1 summarizes the general characteristics of participants across the five study groups (LC, MD, HP, LCK, and control). The groups were comparable in terms of age and BMI, with no statistically significant differences observed. Similarly, there were no significant differences in sex distribution, educational level, employment status, or income categories among the groups (all *p* > 0.05), indicating good homogeneity. The duration of adherence to the dietary regimen also did not differ significantly between groups (*p* = 0.10). The majority of the participants were following dietary regimen for more than 3 months and 55% of MD group were following dietary regimen for more than 6 months.

**Table 1:** General characteristics of the studied groups.

Variables	(LC) (*n* = 40)	(MD) (*n* = 40)	(HP) (*n* = 40)	(LCK) (*n* = 40)	(Control) (*n* = 40)	*F*	*p*
Age (years)	30.15 ± 5.63	30.78 ± 5.31	27.95 ± 7.25	31.68 ± 5.52	29.03 ± 6.86	2.25	0.07
BMI (Kg/m^2^)	26.33 ± 4.43	25 ± 3.92	24.75 ± 5.05	27.04 ± 4.74	24.19 ± 6.14	2.31	0.06

Variables	No	%	No	%	No	%	No	%	No	%	*χ* ^2^	*p*
Sex												
Male	20	50	24	60	24	60	17	42.5	17	45	4.32	0.36
Female	20	50	16	40	16	40	23	57.5	23	55		
Education												
Low	2	5	0	0	5	12.5	4	10	3	7.5	12.39	0.42
Secondary	2	5	1	2.5	3	7.5	0	0	3	7.5		
University	29	72.5	35	87.5	27	67.5	33	82.5	30	75		
Postgraduate	7	17.5	4	10	5	12.5	3	7.5	4	10		
Occupation												
Not working	11	27.5	7	17.5	15	37.5	7	17.5	12	30	6.13	0.19
Working	29	72.5	33	82.5	25	62.5	33	82.5	28	70		
Income												
< 3,000	0	0	0	0	2	5	1	2.5	2	5	27.84	0.11
3,000- < 8,000	5	12.5	1	2.5	2	5	1	2.5	5	12.5		
8,000- < 13,000	6	15	0	0	4	10	2	5	4	10		
13,000–20,000	2	5	7	17.5	7	17.5	10	25	6	15		
>20,000	18	45	22	55	14	35	21	52.5	13	32.5		
Not answer	9	22.5	10	25	11	27.5	5	12.5	10	25		
Duration of regimen (months)												
1–3 m	5	12.5	0	0	1	2.5	4	10	---	---	10.78	0.10
3–6 m	22	55	18	45	18	45	26	65				
>6 m	13	32.5	22	55	21	52.5	10	25				

LC, Low-calorie diet; MD, Mediterranean diet; HP, High protein; LCK, Low-calorie ketogenic diet.

Data presented as mean ±SD or Number and %, F, ANOVA test; *χ*^2^, Chi square test.

### 3.2 Energy and macronutrient intake among the studied groups

Table 2 presents the average daily energy intake and macronutrient distribution (fat, protein, and carbohydrates, listed as percentage of total energy) among participants on different diets. There were significant differences between the diet groups for total calorie intake and macronutrient composition (*p* < 0.001). The LCK group demonstrated a markedly lower caloric (1186.78 ± 231.4 kcal/day) and carbohydrate intake (6.24% ± 2.19%) and a substantially higher proportion of fat intake (66.06% ± 4.7%) compared with other groups, while the HP group showed the highest protein contribution with 33.5% ± 3.69%.

**Table 2:** Energy and macronutrient intake across the different studied groups.

Variable	(LC) (*n* = 40)	(MD) (*n* = 40)	HP (*n* = 40)	LCK (*n* = 40)	Control (*n* = 40)	F/KW	*p*
Calorie intake (kcal)	1764.25 ± 329.04	1925.3 ± 268.8a	2143.08 ± 307a,b	1186.78 ± 231.4a,b,c	1915.75 ± 355.1a,c,d	57.5	<0.001^**^
Fat (%)	31.04 ± 11.7	28.13 ± 4.9	27.05 ± 5.79a	66.06 ± 4.7a,b,c	33.11 ± 4.66b,c,d	197	<0.001^**^
Protein (%)	26.54 ± 2.6	23.06 ± 2.77	33.5 ± 3.96	27.71 ± 3.83	17.28 ± 4.68	87.2	<0.001^**^
Carbohydrate (%)	44.29 ± 5.45	48.75 ± 3.02	40.37 ± 7.28	6.24 ± 2.19	49.31 ± 7.43	304	<0.001^**^

LC, Low-Calorie diet; MD, Mediterranean diet; HP, High protein; LCK, Low-Calorie Ketogenic diet.

Data presented as mean ±SD (Stander deviation), or Number and %, F, ANOVA test; KW, Kruskal Wallis test; *χ*^2^, Chi square test; *, Significant (*p* < 0.05); **, Highly significant (*p* < 0.001); Post hoc, LSD for ANOVA and Dunn’s test for KW; a, Significant versus LC Group; b, Significant versus MD Group; c, Significant versus HP Group; d, Significant versus LCK Group.

### 3.3 Sleep behavior among the studied groups

Table 3 reveals that there were statistically significant differences in PSQI scores among the studied groups (*p* < 0.001). The results indicated that the PSQI score among the MD group was the lowest (score 3), which indicated the best sleep quality. In contrast, the LCK group had the highest PSQI score (score 8), which indicated the worst sleep quality compared to other groups. Furthermore, in terms of categorical analysis, a significantly higher proportion of participants in the MD group reported good sleep quality (95%) was observed, whereas the LCK group had the highest prevalence of poor sleep quality (90%) (*χ*^2^ = 78.88, *p* < 0.001). Regarding sleep duration, most participants in the MD group reported normal sleep duration (7–8 h) (92.5%), followed by the HP group (52.5%). Conversely, short sleep duration (≤6 h) was most prevalent in the LCK and control groups (72.5 and 82.5%, respectively). Additionally, the proportion of participants reporting that stress or personal problems affected their sleep differed significantly across groups (*χ*^2^ = 33.81, *p* < 0.001). None of the MD participants reported stress-related sleep disturbances, whereas more than half of the control group (57.5%) and nearly one-third of the LCK group (32.5%) reported that stress negatively affected their sleep.

**Table 3:** Sleep behavior among the studied groups.

Variable	(LC) (*n* = 40)	(MD) (*n* = 40)	(HP) (*n* = 40)	(LCK) (*n* = 40)	(Control) (*n* = 40)	KW	*p*
Subjective sleep quality	1(0–3)	0 (0–1) a	1 (0–2) b	1 (0–3) b	1 (0–3) b	50.8	<0.001**
Sleep latency	2 (1–3)	1 (0–2) a	1 (0–3)	2(0–3) b	1.5 (0–3)	47.6	<0.001**
Sleep duration	1 (1–3)	1 (1) a	1 (1–2) b	1 (1–3) b	1 (1–3) b	18.8	0.001*
Habitual sleep efficiency	0 (0–3)	0 (0–3)	0.5 (0–3)	0 (0–3)	0 (0–3)	5.97	0.20
Sleep disturbances	1 (0–2)	1(0–1) a	1 (0–2) b	1 (1–2) b	1 (0–3) b	11.5	0.02*
Sleep Medication	0 (0–2)	0 (0–1)	0 (0–3)	0.5 (0–2)	0 (0–3)	7.6	0.09
Day time dysfunction	1 (0–3)	0 (0–2) a	0.5 (0–2)	1 (0–3) b	1 (0–3) b	55.4	<0.001**
Total PSQI	7 (4–13)	3 (0–9) a	5 (2–15) a,b	8 (3–16) b,c	7.5 (2–15) b,c	73.4	<0.001**

Variables	No	%	No	%	No	%	No	%	No	%	*χ* ^2^	*p*
Sleep quality based on PSQI												
Good	8	20	38	95	22	55	4	10	10	25	78.88	<0.001**
Poor	32	80	2	5	18	45	36	90	30	75		
Sleep duration												
Very short (<5 h)	4	10	0	0	2	5	3	7.5	9	22.5	73.67	<0.001**
Short (5–6 h)	18	45	0	0	16	40	26	65	24	60		
Normal (7–8 h)	17	42.5	37	92.5	21	52.5	11	27.5	5	12.5		
Long (≥9 h)	1	2.5	3	7.5	1	2.5	0	0	2	5		

Data presented as median (range) or Number and %, F, ANOVA test; KW, Kruskal Wallis test; *χ*^2^, Chi square test; *, Significant (*p* < 0.05); **, Highly significant (*p* < 0.001); Post hoc, LSD for ANOVA and Dunn’s test for KW; a, Significant versus LC Group; b, Significant versus Group MD; c, Significant versus Group HP; d, Significant versus LCK Group.

### 3.4 Level of physical activity among the studied group

Table 4 presents the distribution of physical activity levels and timing across the studied dietary groups. A significant difference was found in physical activity levels among the groups (*χ*^2^ = 85.61, *p* < 0.001). The HP group had the highest percentage of highly active participants at 80%. Meanwhile, the control group showed the highest rate of inactivity at 60%. The MD and LC groups were mainly minimally active (55% and 50%, respectively).

**Table 4:** Physical activity levels among the studied groups.

Variables	(LC) (*n* = 40)		(MD) (*n* = 40)		(HP) (*n* = 40)		(LCK) (*n* = 40)		(Control) (*n* = 40)		χ^2^	*p*
	No	%	No	%	No	%	No	%	No	%		
Physical activity												
Inactive	2	5	9	22.5	2	5	17	42.5	24	60	85.61	<0.001 **
Moderate active	20	50	22	55	6	15	19	47.5	4	10		
High active	18	45	9	22.5	32	80	4	10	12	30		

Data presented as Number and % *χ*^2^: Chi square test, **: Highly significant (*p* < 0.05).

### 3.5 Sleep quality and physical activity

Table 5 showed that there was a statistically significant difference in sleep quality in relation to physical activity levels (*p* = 0.001). Among inactive participants, only 20.4% (*n* = 11) reported good sleep quality. In contrast, 50.7% (*n* = 36) of the moderate active group reported good sleep quality, followed by the high active group (46.7%). This finding indicated that higher physical activity levels were significantly associated with better sleep quality, while inactive participants were more likely to have poor sleep quality. The association between sleep duration and physical activity levels was statistically significant (*p* = 0.009). Inactive participants had a considerably higher frequency of short sleep 53.7% (*n* = 29), while 62% (*n* = 44) of moderate active participants reported normal sleep duration. This result indicated that moderate active participants were significantly associated with longer hours of sleep, while inactive participants being more likely to have short sleep duration (Table 5).

**Table 5:** Physical activity levels by sleep quality and duration.

Variables	Physical activity levels			χ^2^	*p*
	Inactive *N* (%)	Moderate active *N* (%)	High active *N* (%)		
Sleep quality					
Good (*n* = 82)	11 (20.4)	36 (50.7)	35 (46.7)	13.26	0.001*
Poor (*n* = 118)	43 (79.6)	35 (49.3)	40 (53.3)		
Sleep duration					
Very short (*n* = 18)	7 (13.0)	2 (2.8)	9 (12.0)	17.03	0.009*
Short (*n* = 48)	29 (53.7)	23 (32.4)	32 (42.7)		
Normal (*n* = 91)	15 (27.8)	44 (62.0)	32 (42.7)		
Long (*n* = 7)	3 (5.6)	2 (2.8)	2 (2.7)		

*χ*^2^: Chi square test, *: Significant (*p* < 0.05).

## 4 Discussion

This study examined whether clients in diet programs in Jeddah, Saudi Arabia adopt a healthy lifestyle beyond their diet plans. It specifically investigated if they integrate adequate physical activity and maintain good sleep habits alongside dietary changes. The findings reveal a complex pattern of lifestyle adoption that varies according to different diets. The type of diet was significantly associated with non-diet behaviors. Some diets led to positive lifestyle outcomes, while others were related to unhealthy lifestyle behaviors, particularly concerning sleep.

Our results indicate that the various diet groups (LC, MD, HP, and LCK) and the control group were well-matched in key demographic and general characteristics. This homogeneity strengthens the internal validity of the study, allowing for more confident interpretation of observed differences in sleep and physical activity to the dietary interventions or lifestyle patterns rather than confounding variables (34). The significant differences in energy and macronutrient intake confirm that the groups adhered to distinct dietary plans as prescribed by the diet centers, supporting the study’s design for comparing lifestyle outcomes across different nutritional approaches.

A key finding of this study is the obvious difference in sleep quality and duration among diet groups. Participants following the MD diet showed the healthiest sleep patterns, since they obtained the lowest median PSQI score, which is an indication of the best sleep quality. Additionally, 95% of them reported good sleep, and 92.5% achieved a normal sleep duration of 7–8 h. This aligns with other research that associates healthy diets rich in vegetables, fruits, and whole grains with better sleep (13, 35). Notably, none of the Mediterranean diet participants reported sleep problems caused by stress. This may relate to nutrient composition of the diet including higher intakes of magnesium and tryptophan, which are key for sleep regulation (36). Tryptophan supports sleep by boosting serotonin and melatonin production that regulate the sleep–wake cycle. Additionally, magnesium enhancing neurotransmitter activity that promotes deeper sleep (37). Observational studies show that adequate magnesium levels are linked to fewer awakenings, longer sleep duration, and less daytime sleepiness (38).

The pattern of physical activity also varied significantly by diet group. The HP group had 80% classified as “high active,” representing the highest proportion among all groups. This suggests that individuals prescribed a HP diet in our sample may be more likely to engage in, or perhaps are prescribed, concomitant exercise, as protein intake is often emphasized in conjunction with resistance training for body composition goals (39). Interestingly, the LCK group had a high percentage of inactive individuals (42.5%), coupled with their poor sleep (90%), which presents a concerning profile. This association suggests that the adherence to ketogenic diet, whether behavioral or physiological, may coexist with lower regular physical activity for a substantial portion of adherents. The ketogenic diet may alter sleep physiology by modifying brain energy metabolism, which can reduce night-time awakenings in some individuals. In addition, reduced carbohydrate intake may lower tryptophan availability, potentially disrupting melatonin synthesis, contributing to possible sleep dysregulation for certain individuals (40). This finding highlights the need for further investigation.

Of note, as with physical activity levels, our data also show a highly positive correlation between physical activity levels and sleep quality (even at the individual level) as more active participants were statistically significantly more likely to report good quality sleep and to report normal sleep duration. Routine physical activity improves sleep onset latency, duration, and quality and increased sleep facilitates exercise performance and recovery (41, 42), making this bidirectional relationship well-established. However, the observation that the MD group demonstrated superior sleep outcomes despite being only minimally active, whereas the highly active HP group showed only intermediate sleep quality, suggests that dietary objectives and composition may differentially influence sleep health. HP diets are often adopted to support muscle development and performance, frequently accompanied by intensive training and higher physiological stress, which may attenuate sleep quality despite high activity levels (43). In contrast, the MD is widely conceptualized as a rebalancing dietary pattern grounded in moderation, dietary diversity, and circadian-friendly nutrient profiles (44), which have been consistently associated with better sleep quality and duration independent of physical activity (13). These findings indicate that while physical activity remains a cornerstone of a healthy lifestyle, the underlying dietary perspective (performance-oriented versus holistic and moderate) was independently associated with sleep health in the present study.

The MD group, while adhering to their Centre’s prescribed dietary pattern, had a mean energy intake (1925 kcal) that was not calorically restricted compared to other groups. This, along with their superior sleep outcomes, suggests their primary motivation for enrolling may extend beyond weight loss. Their profile may reflect a growing demographic in Saudi Arabia seeking structured dietary programs for general wellness, disease prevention, and holistic lifestyle improvement (45). This trend is actively encouraged by national public health initiatives promoting comprehensive healthy lifestyles and is a significant driver in the Kingdom’s rapidly expanding health and wellness market (46). Future research should collect explicit data on enrolment motivations and perform detailed sociodemographic comparisons to test if clients choosing Mediterranean-style plans differ systematically in income, education, or health literacy from those opting for weight-loss-focused regimens like LC or LCK.

This study has several limitations. Its cross-sectional design precludes causal inference. Therefore, observed associations should be interpreted cautiously and cannot establish temporal or causal relationships. In addition, data on dietary adherence, physical activity, and sleep were self-reported, which are subject to recall and social desirability biases. The sample was drawn from clients of diet centers in one region of Saudi Arabia, which may limit generalizability to other populations or to individuals pursuing weight management independently. As for the control group, it was recruited via social media advertisements, which may have introduced selection bias, as participants who self-select into online surveys may differ systematically in health awareness, motivation, and lifestyle behaviors from the broader population. Despite these limitations, the study’s strengths include its direct comparison of multiple popular diets, the use of validated tools (PSQI and IPAQ), and the examination of the interconnection between two critical lifestyle components often ignored in dietary efficacy studies. Finally, it is worth mentioning that given the exploratory design of this study, interpretation focused primarily on statistically significant findings while also considering the direction and magnitude of observed differences to avoid over-interpretation of non-robust associations.

## 5 Conclusion

Although clients of diet centers generally adhere to prescribed dietary plans, adoption of complementary healthy behaviors such as adequate physical activity and good sleep quality is inconsistent. The Mediterranean diet model was associated with better sleep health, consistent with its nutritional profile that supports sleep physiology. The LCK diet was associated with significantly poorer sleep and a trend towards physical inactivity in this cross-sectional study, suggesting potential short-term challenges despite evidence of long-term benefits via weight loss. High-protein diets were strongly linked to higher physical activity levels. The significant association between physical activity and better sleep reinforces the observed interconnectedness of lifestyle behaviors. Therefore, diet centers are encouraged to integrate structured sleep hygiene education and personalized physical activity guidance into their programs to foster truly comprehensive wellness.

## Data Availability

The original contributions presented in the study are included in the article/supplementary material, further inquiries can be directed to the corresponding author.

## References

[1] Mariod A Tahir HE Salama S. "Food consumption patterns in Saudi Arabia". In: Ahmed AE Al-Khayri JM Elbushra AA, editors. Food and Nutrition Security in the Kingdom of Saudi Arabia. Cham: Springer (2024). doi: 10.1007/978-3-031-46704-2_13

[2] Al-Omar HA Alshehri A Alqahtani SA Alabdulkarim H Alrumaih A Eldin MS. A systematic review of obesity burden in Saudi Arabia: prevalence and associated co-morbidities. Saudi Pharm J. (2024) 32:102192. doi: 10.1016/j.jsps.2024.102192, 39525490 PMC11550078

[3] GASTAT. (2024). Health determinants statistics publication in Saudi Arabia 2024. Available online at: https://www.stats.gov.sa/en/w/ [Accessed January 5, 2025].

[4] Ahmed SK Mohammed RA. Obesity: prevalence, causes, consequences, management, preventive strategies and future research directions. Metabol Open. (2025) 27:100375. doi: 10.1016/j.metop.2025.100375, 41041606 PMC12486175

[5] Browne S Minozzi S Bellisario C Sweeney MR Susta D. Effectiveness of interventions aimed at improving dietary behavior’s among people at higher risk of or with chronic non-communicable diseases: an overview of systematic reviews. Eur J Clin Nutr. (2019) 73:9–23. doi: 10.1038/s41430-018-0327-3, 30353122

[6] Onu A Trofin DM Tutu A Onu I Galaction AI Sardaru DP . Integrative strategies for preventing and managing metabolic syndrome: the impact of exercise and diet on oxidative stress reduction—a review. Life. (2025) 15:757. doi: 10.3390/life15050757, 40430185 PMC12113156

[7] Johns DJ Hartmann-Boyce J Jebb SA Aveyard P Behavioral Weight Management Review Group. Diet or exercise interventions vs combined behavioral weight management programs: a systematic review and meta-analysis of direct comparisons. J Acad Nutr Diet. (2014) 114:1557–68. doi: 10.1016/j.jand.2014.07.005, 25257365 PMC4180002

[8] Pojednic R D’Arpino E Halliday I Bantham A. The benefits of physical activity for people with obesity, independent of weight loss: a systematic review. Int J Environ Res Public Health. (2022) 19:4981. doi: 10.3390/ijerph19094981, 35564376 PMC9102424

[9] Cox CE. Role of physical activity for weight loss and weight maintenance. Diabetes Spectr. (2017) 30:157–60. doi: 10.2337/ds17-0013, 28848307 PMC5556592

[10] Knutson KL Van Cauter E. Associations between sleep loss and increased risk of obesity and diabetes. Ann N Y Acad Sci. (2008) 1129:287–304. doi: 10.1196/annals.1417.033, 18591489 PMC4394987

[11] St-Onge MP Mikic A Pietrolungo CE. Effects of diet on sleep quality. Adv Nutr. (2016) 7:938–49. doi: 10.3945/an.116.012336, 27633109 PMC5015038

[12] Jansen EC Prather A Leung CW. Associations between sleep duration and dietary quality: results from a nationally-representative survey of US adults. Appetite. (2020) 153:104748. doi: 10.1016/j.appet.2020.104748, 32442470

[13] Godos J Grosso G Castellano S Galvano F Caraci F Ferri R. Association between diet and sleep quality: a systematic review. Sleep Med Rev. (2021) 57:101430. doi: 10.1016/j.smrv.2021.101430, 33549913

[14] Bacaro V Ballesio A Cerolini S Vacca M Poggiogalle E Donini LM . Sleep duration and obesity in adulthood: an updated systematic review and meta-analysis. Obes Res Clin Pract. (2020) 14:301–9. doi: 10.1016/j.orcp.2020.03.004, 32527625

[15] Kohanmoo A Akhlaghi M Sasani N Nouripour F Lombardo C Kazemi A. Short sleep duration is associated with higher risk of central obesity in adults: a systematic review and meta-analysis of prospective cohort studies. Obes Sci Pract. (2024) 10:e772. doi: 10.1002/osp4.772, 38835720 PMC11149606

[16] Alqahtani BA Alenazi AM Alhowimel AS Elnaggar RK. The descriptive pattern of physical activity in Saudi Arabia: analysis of national survey data. Int Health. (2021) 13:232–9. doi: 10.1093/inthealth/ihaa027, 32511710 PMC8079316

[17] Lee IM Shiroma EJ Lobelo F Puska P Blair SN Katzmarzyk PT. Effect of physical inactivity on major non communicable diseases worldwide: an analysis of burden of disease and life expectancy. Lancet. (2012) 380:219–29. doi: 10.1016/S0140-6736(12)61031-9, 22818936 PMC3645500

[18] Al Muammar S Algarni A Sadi RA Mushaeb H Badroun F Algarni A. Sleep patterns and problems among adults in Saudi Arabia: a cross-sectional survey-based study. Med Sci. (2023) 27:e308ms3122. doi: 10.54905/disssi/v27i137/e308ms3122

[19] Almutairi KM Alonazi WB Vinluan JM Almigbal TH Batais MA Alodhayani AA . Health promoting lifestyle of university students in Saudi Arabia: a cross-sectional assessment. BMC Public Health. (2018) 18:1093. doi: 10.1186/s12889-018-5999-z 30185167 PMC6126031

[20] Al-Hazzaa HM. Physical inactivity in Saudi Arabia revisited: a systematic review of inactivity prevalence and perceived barriers to active living. Int J Health Sci. (2018) 12:50–64. PMC6257875 30534044

[21] AlTamimi AA Albawardi NM AlMarzooqi MA Aljubairi M Al-Hazzaa HM. Lifestyle behaviors and socio-demographic factors associated with overweight or obesity among Saudi females attending fitness centers. Diabetes Metab Syndr Obes. (2020) 13:2613–22. doi: 10.2147/DMSO.S255628 32821137 PMC7419638

[22] Fatima Y Mamun AA Skinner T. "Association between obesity and poor sleep: a review of epidemiological evidence". In: Pathophysiology of Obesity-Induced Health Complications. Cham: Springer (2020). doi: 10.1007/978-3-030-35358-2_9

[23] Carter R III Watenpaugh DE. Obesity and obstructive sleep apnea: or is it OSA and obesity? Pathophysiology. (2008) 15:71–7. doi: 10.1016/j.pathophys.2008.04.009 18573643

[24] Chaput JP Després JP Bouchard C Tremblay A. Short sleep duration is associated with reduced leptin levels and increased adiposity: results from the Quebec family study. Obesity. (2007) 15:253–61. doi: 10.1038/oby.2007.512 17228054

[25] St-Onge MP Grandner MA Brown D Conroy MB Jean-Louis G Coons M . Sleep duration and quality: impact on lifestyle behaviors and cardio metabolic health: a scientific statement from the American Heart Association. Circulation. (2016) 134:e367–86. doi: 10.1161/CIR.0000000000000444, 27647451 PMC5567876

[26] Ekkekakis P Lind E. Exercise does not feel the same when you are overweight: the impact of self-selected and imposed intensity on affect and exertion. Int J Obes. (2006) 30:652–60. doi: 10.1038/sj.ijo.0803052 16130028

[27] Buysse DJ Reynolds CF III Monk TH Berman SR Kupfer DJ. The Pittsburgh sleep quality index: a new instrument for psychiatric practice and research. Psychiatry Res. (1989) 28:193–213. doi: 10.1016/0165-1781(89)90047-4, 2748771

[28] Helou K El Helou N Mahfouz M Mahfouz Y Salameh P Harmouche-Karaki M. Validity and reliability of an adapted Arabic version of the long international physical activity questionnaire. BMC Public Health. (2017) 18:49. doi: 10.1186/s12889-017-4599-7, 28738790 PMC5525276

[29] Komaroff M. For researchers on obesity: historical review of extra body weight definitions. J Obes. (2016) 2016:2460285. doi: 10.1155/2016/2460285, 27313875 PMC4904092

[30] Suleiman KH Yates BC Berger AM Pozehl B Meza J. Translating the Pittsburgh sleep quality index into Arabic. West J Nurs Res. (2010) 32:250–68. doi: 10.1177/0193945909348230, 19915205

[31] Sjostrom M Ainsworth BE Bauman A Bull FC Hamilton-Craig CR Sallis JF (2005) Guidelines for data processing analysis of the international physical activity questionnaire (IPAQ)—Short and long forms. Available online at: http://www.ipaq.ki.se/scoring.pdf (Accessed February 28, 2025).

[32] Shamah-Levy T Rodríguez-Ramírez S Gaona-Pineda EB Cuevas-Nasu L Carriquiry AL Rivera JA. Three 24-hour recalls in comparison with one improve the estimates of energy and nutrient intakes in an urban Mexican population. J Nutr. (2016) 146:1043–50. doi: 10.3945/jn.115.219683, 27052536

[33] Baranowski T. "24-hour recall and diet record methods". In: Nutritional Epidemiology. New York: Oxford (2013). doi: 10.1093/acprof:oso/9780199754038.003.0004

[34] Ferguson L. External validity, generalizability, and knowledge utilization. J Nurs Scholarsh. (2004) 36:16–22. doi: 10.1111/j.1547-5069.2004.04006.x 15098414

[35] Abou-Khalil R. Nutritional interventions for enhancing sleep quality: the role of diet and key nutrients in regulating sleep patterns and disorders. Food Sci Nutr. (2025) 13:e71309. doi: 10.1002/fsn3.71309, 41356231 PMC12678061

[36] Scoditti E Tumolo MR Garbarino S. Mediterranean diet on sleep: a health alliance. Nutrients. (2022) 14:2998. doi: 10.3390/nu14142998, 35889954 PMC9318336

[37] Arab A Rafie N Amani R Shirani F. The role of magnesium in sleep health: a systematic review of available literature. Biol Trace Elem Res. (2023) 201:121–8. doi: 10.1007/s12011-022-03162-1, 35184264

[38] Schmitt F Weishaupt R Katumba P Vogt D Meyer N Feld M . Improving sleep and daytime function with tryptophan, magnesium, Melissa and Lactuca formulation: an exploratory study in adults with sleep disturbances. Nutr Diet Suppl. (2025) 17:75–85. doi: 10.2147/NDS.S550592

[39] Jäger R Kerksick CM Campbell BI Cribb PJ Wells SD Skwiat TM . International society of sports nutrition position stand: protein and exercise. J Int Soc Sports Nutr. (2017) 14:20. doi: 10.1186/s12970-017-0177-8, 28642676 PMC5477153

[40] Kouki N. How effective is ketogenic diet in sleep disorders? Eur Psychiatry. (2024) 67:1617. doi: 10.1192/j.eurpsy.2024.1617

[41] Kredlow MA Capozzoli MC Hearon BA Calkins AW Otto MW. The effects of physical activity on sleep: a meta-analytic review. J Behav Med. (2015) 38:427–49. doi: 10.1007/s10865-015-9617-6, 25596964

[42] Venter RE. Role of sleep in performance and recovery of athletes: a review article. S Afr J Res Sport Phys Educ Recreation. (2012) 34:167–84. doi: 10.4314/sajrs.v34i1

[43] Alnawwar MA Alraddadi MI Algethmi RA Salem GA Salem MA Alharbi AA. The effect of physical activity on sleep quality and sleep disorder: a systematic review. Cureus. (2023) 15:e43595. doi: 10.7759/cureus.43595, 37719583 PMC10503965

[44] Real H Queiroz J Graca P. Mediterranean food pattern *vs.* Mediterranean diet: a necessary approach? Int J Food Sci Nutr. (2020) 71:1–12. doi: 10.1080/09637486.2019.1617838, 31122086

[45] IMARC Group. (2023). Saudi Arabia health and wellness market: Industry trends, share, size, growth, opportunity and forecast 2023–2028 (Report No. SR112023A7095). Available online at: https://www.imarcgroup.com/saudi-arabia-health-wellness-market [Accessed February 7, 2026].

[46] Kingdom of Saudi Arabia, Ministry of Health. (2025). Awareness platform: Healthy lifestyle. Available online at: https://www.moh.gov.sa/en/awarenessplateform/healthylifestyle/pages/default.aspx [Accessed February 7, 2026].

